# Exopolyhedral Ligand Orientation Controls Diastereoisomer in Mixed-Metal Bis(Carboranes) †

**DOI:** 10.3390/molecules25030519

**Published:** 2020-01-24

**Authors:** Antony P. Y. Chan, Georgina M. Rosair, Alan J. Welch

**Affiliations:** Institute of Chemical Sciences, School of Engineering & Physical Sciences, Heriot-Watt University, Edinburgh EH14 4AS, UK; A.P.Y.Chan@liverpool.ac.uk (A.P.Y.C.); g.m.rosair@hw.ac.uk (G.M.R.)

**Keywords:** carborane, metallacarborane, diastereoisomer, steric crowding

## Abstract

Heterobimetallic derivatives of a bis(carborane), [μ_7,8_-(1′,3′−3′-Cl-3′-PPh_3_-*closo*-3′,1′,2′-RhC_2_B_9_H_10_)-2-(*p*-cymene)-*closo*-2,1,8-RuC_2_B_9_H_10_] (**1**) and [μ_7,8_-(1′,3′−3′-Cl-3′-PPh_3_-*closo*-3′,1′,2′-RhC_2_B_9_H_10_)-2-Cp-*closo*-2,1,8-CoC_2_B_9_H_10_] (**2**) have been synthesised and characterised, including crystallographic studies. A minor co-product during the synthesis of compound **2** is the new species [8-{8′-2′-H-2′,2′-(PPh_3_)_2_-*closo*-2′,1′,8′-RhC_2_B_9_H_10_}-2-Cp-*closo*-2,1,8-CoC_2_B_9_H_10_] (**3**), isolated as a mixture of diastereoisomers. Although, in principle, compounds **1** and **2** could also exist as two diastereoisomers, only one (the same in both cases) is formed. It is suggested that the preferred exopolyhedral ligand orientation in the rhodacarboranes in the non-observed diastereoisomers would lead to unacceptable steric crowding between the PPh_3_ ligand and either the *p*-cymene (compound **1**) or Cp (compound **2**) ligand of the ruthenacarborane or cobaltacarborane, respectively.

## 1. Introduction

**Scheme 1:**
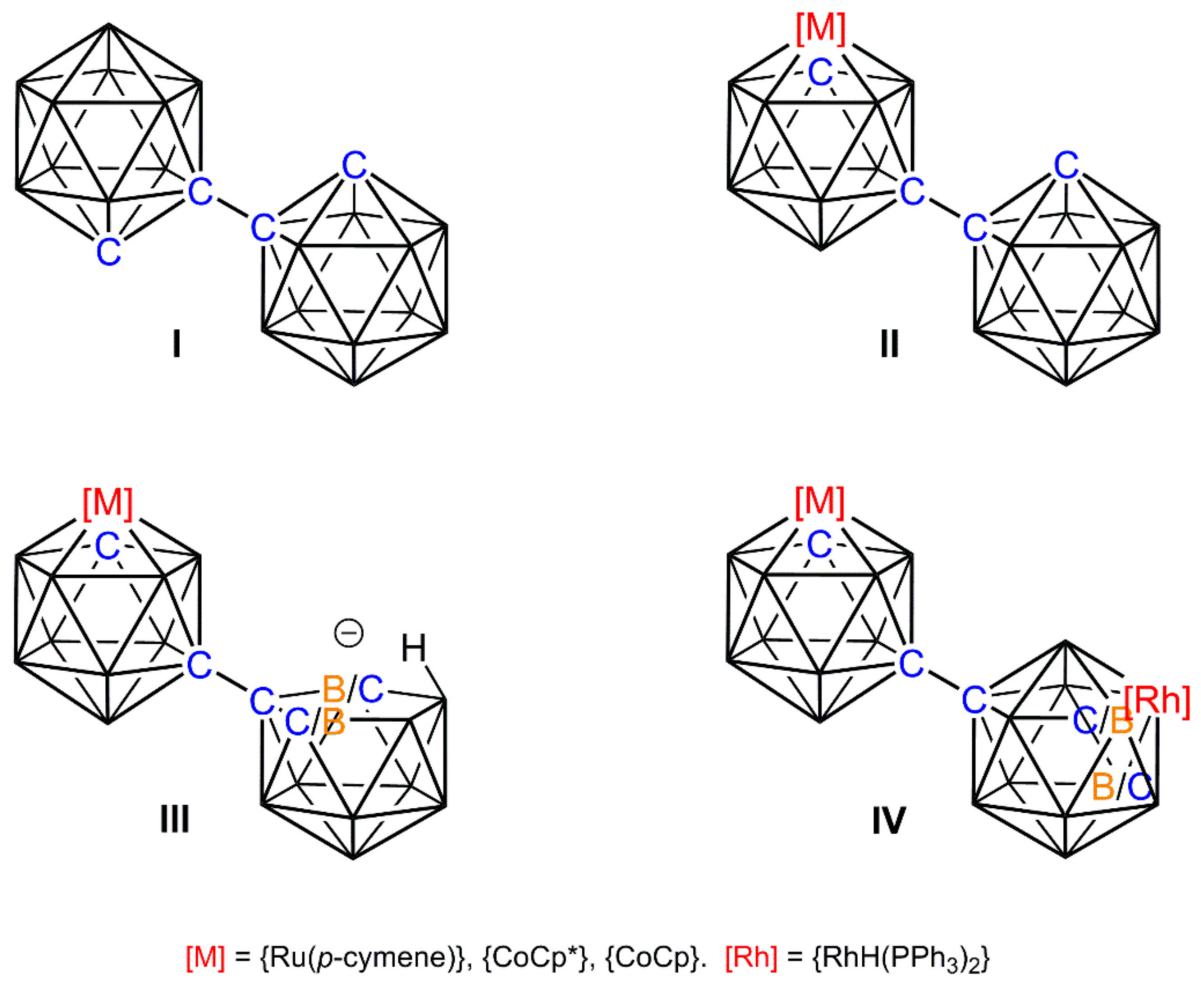
Line diagrams of **I**–**IV**. Species **III** and **IV** exist as diastereoisomers in which the second cage C atom could either be at the vertex labelled C/B or the vertex labelled B/C. Unlabelled vertices are B.

Recent years have witnessed a significant amount of interest in the chemistry of bis(carboranes) [1,2,3,4], particularly 1,1′-bis(*ortho*-carborane), formally [1-(1′-*closo*-1′,2′-C_2_B_10_H_11_)-*closo*-1,2-C_2_B_10_H_11_] (**I**), Scheme 1. This species offers a versatile scaffold for derivatisation and, amongst other studies, we have reported the single deboronation/metalation [5], double deboronation/homometalation [6] and stepwise deboronation/metalation-deboronation/heterometalation [7] of **I**, the latter including the use of {Rh(H)(PPh_3_)_2_} fragments to afford homogeneous catalyst precursors [8].

Specifically, [8-(1′-*closo*-1′,2′-C_2_B_10_H_11_)-2-L-*closo*-2,1,8-MC_2_B_9_H_10_] [**II**, M = Ru, L = (*p*-cymene); M = Co, L = Cp*] was deboronated to afford the anion [8-(7′-*nido*-7′,8′-C_2_B_19_H_11_)-2-L-*closo*-2,1,8-MC_2_B_9_H_10_]^−^ (**III**) as a mixture of diastereoisomers. Deprotonation of the endo H atom and reaction with [Rh(PPh_3_)_3_Cl] led to isomerisation of the primed cage and the isolation of the product [8-{8′-2′-H-2′,2′-(PPh_3_)_2_-*closo*-2′,1′,8′-RhC_2_B_9_H_11_}-2-L-*closo*-2,1,8-MC_2_B_9_H_10_] (**IV**) as a diastereoisomeric mixture [8]. Compounds of type **IV** were found to be active catalyst precursors for alkene isomerisation and the hydrosilylation of acetophenone.

Seeking to expand the scope of this chemistry, we have now investigated the reactions of **III** [M = Ru, L = (*p*-cymene); M = Co, L = Cp] with [Rh(PPh_3_)_3_Cl] under different conditions and here present the results. Whilst these new reactions also afford compounds of type **IV** as minor co-products, the major species produced are unique heterometalated derivatives of bis(carborane), each isolated in only one of two possible diastereoisomeric forms.

## 2. Results and Discussion

### 2.1. Synthesis and Characterisation of Compound ***1***

We have previously reacted **III** [M = Ru, L = (*p*-cymene); **III**_Ru_] following deprotonation, with [Rh(PPh_3_)_3_Cl] under overnight reflux in tetrahydrofuran (THF) to afford **IV**_Ru_ as a mixture of diastereoisomers with a total yield >50%. Repeating this reaction at room temperature, after workup involving preparative thin-layer chromatography (TLC), resulted in the isolation of a new species **1** as the main product, albeit with a modest yield (11%). Also isolated were trace amounts (<1%) of both diastereoisomers of the known species **IV**_Ru_, identified by multinuclear NMR spectroscopy [8].

The ^1^H NMR spectrum of **1** reveals a number of interesting features. The resonances due to the *p*-cymene ligand demonstrated that the molecule is asymmetric, as expected, but the two multiplets (each due to 2H) for the aromatic protons are at very different chemical shifts, δ 6.40–6.36 and 4.97–4.92 ppm. Moreover, while there is clearly only one PPh_3_ ligand present, the resonances due to it appear as two multiplets, one of which is at a relatively high frequency (δ 7.82–7.75 ppm) and integrates for 5H. These results are consistent with a *p*-cymene ligand locked in conformation and a PPh_3_ ligand in which one ring is in a unique environment. Two C_cage_*H* resonances are present, one at δ 2.68 ppm assigned to the ruthenacarborane cage by analogy with the resonance in **IV**_Ru_ [8], and the other at much higher frequency, δ 5.02 ppm, consistent with a non-isomerised 3,1,2-RhC_2_B_9_ rhodacarborane cage.

**Figure 1:**
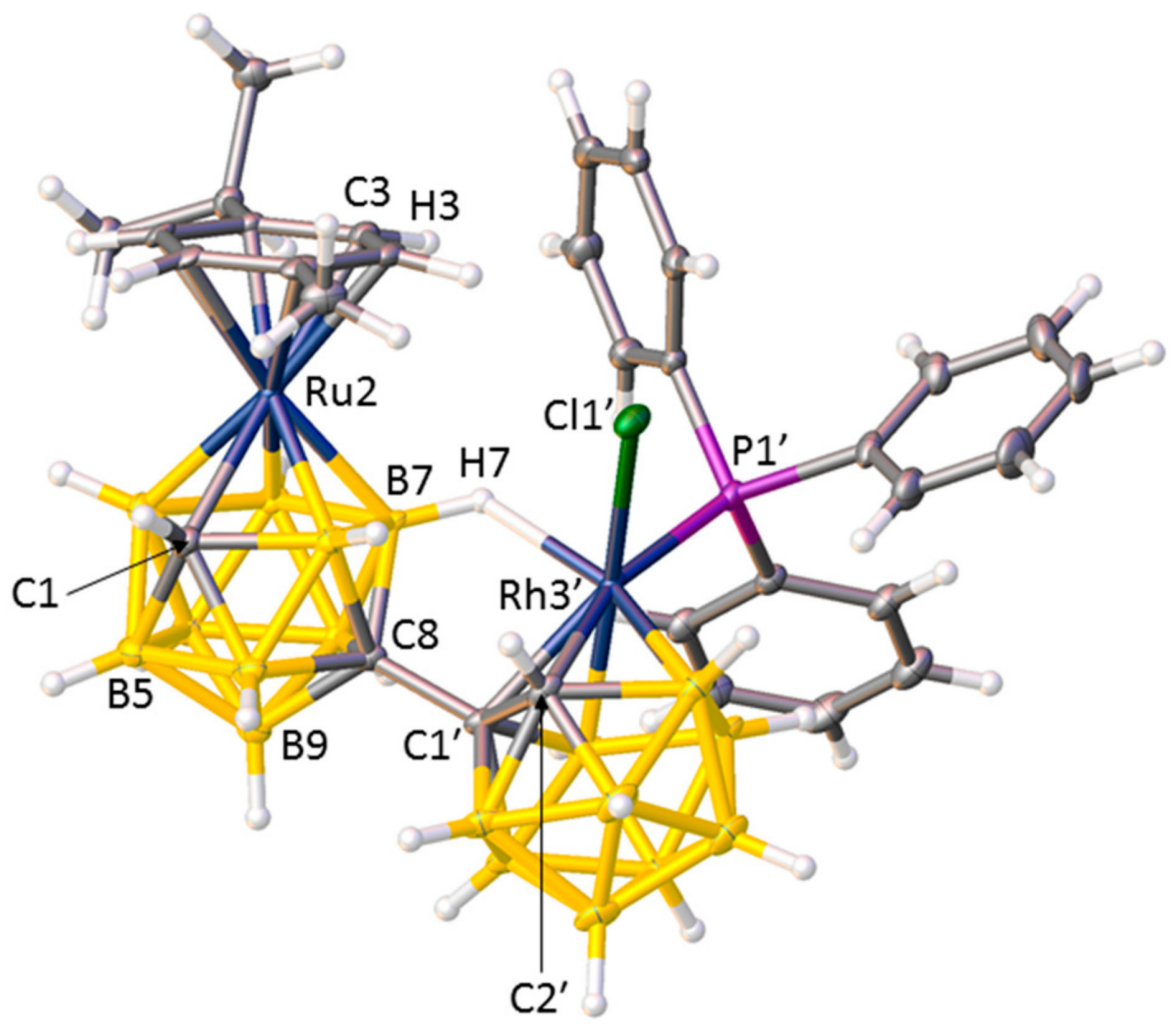
Structure of molecule C, one of three crystallographically independent molecules of compound **1**, with displacement ellipsoids drawn at the 50% probability level except for H atoms. Selected interatomic distances (molecule A, molecule B, molecule C, Å): Ru2−C1 2.173(3), 2.178(4), 2.170(3); C8−C1′ 1.519(4), 1.470(4), 1.532(4); Rh3′−C1′ 2.209(3), 2.190(3), 2.199(3); Rh3′−C2′ 2.210(3), 2.207(3), 2.208(3); Rh3′−Cl1′ 2.4036(7), 2.3689(7), 2.3821(7); Rh3′−P1′ 2.3247(8), 2.3115(8), 2.3133(7); Rh3′−H7 2.03(3), 1.97(3), 2.01(3); C1′−C2′ 1.605(4), 1.603(4), 1.605(4).

A crystallographic study revealed the nature of compound **1**, and Figure 1 shows a perspective view of a single molecule. The open face of the nido cage of deprotonated **III**_Ru_ has been capitated by a {RhCl(PPh_3_)} fragment, and the coordination sphere of the Rh atom is completed by a B7−H7⇀Rh *B*-agostic interaction, a bonding mode observed previously [9]. Importantly the RhC_2_B_9_ cage has not undergone isomerisation. Thus, compound **1** is [μ_7,8_-(1′,3′−3′-Cl-3′-PPh_3_-*closo*-3′,1′,2′-RhC_2_B_9_H_10_)-2-(*p*-cymene)-*closo*-2,1,8-RuC_2_B_9_H_10_].

**Figure 2:**
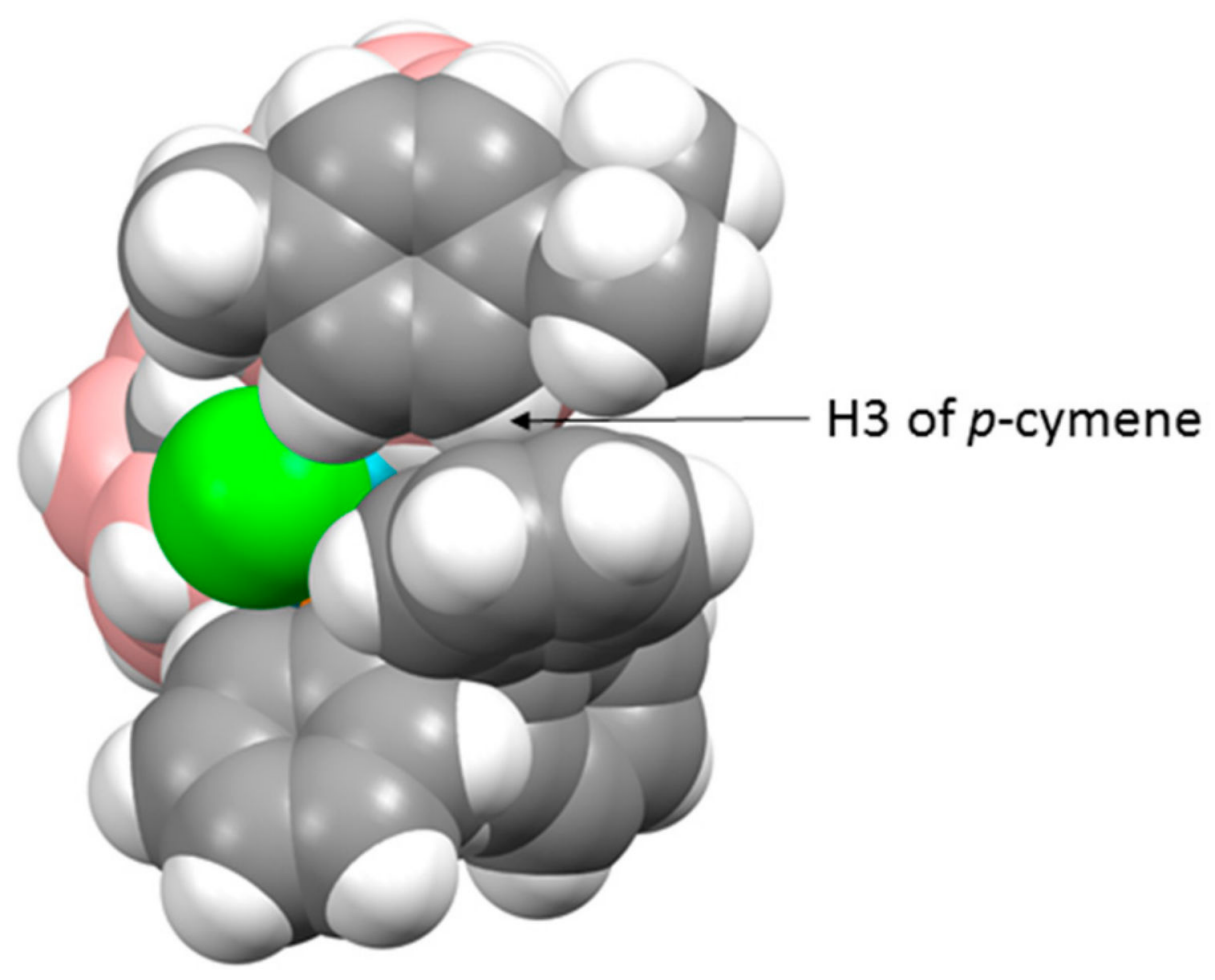
Molecule C of compound **1** viewed from above in space-filling representation.

There are three crystallographically independent molecules of **1** in the asymmetric fraction of the unit cell, a relatively rare occurrence (<0.5% of structures in the Cambridge Crystallographic Database (CCD) [10] have *Z*′ = 3), and all three molecules are practically superimposable. Figure 2 shows a space-filling representation of one molecule viewed from above the *p*-cymene ring. The C3−H3 bond of the *p*-cymene points towards the centre of one of the phenyl rings of the PPh_3_ ligand, with H3 lying only 2.52–2.71 Å from the ring centre, and we believe that this effectively locks both the *p*-cymene ligand and the Ph group in fixed positions even in solution, consistent with the ^1^H NMR spectrum discussed above.

### 2.2. Synthesis and Characterisation of Compounds ***2*** and ***3***

Although deprotonation of **III**_CoCp*_ with *^n^*BuLi followed by treatment with [Rh(PPh_3_)_3_Cl] leads to the heterobimetallic **IV**_CoCp*_ as a mixture of diastereoisomers [8], the same approach cannot be used with the Cp analog **III**_CoCp_ because of the attack on the Cp ring by *^n^*BuLi. Accordingly, we have reverted to direct reaction between [HNMe_3_][8-(7′-*nido*-7′,8′-C_2_B_19_H_11_)-2-Cp-*closo*-2,1,8-CoC_2_B_9_H_10_] and [Rh(PPh_3_)_3_Cl] under reflux in ethanol, mimicking Hawthorne’s original synthesis of the classic rhodacarborane catalyst [3-H-3,3-(PPh_3_)_2_-*closo*-3,1,2-RhC_2_B_9_H_11_] [11]. This synthetic approach led to the isolation of two new products following workup. The minor product, isolated as an inseparable mixture of diastereoisomers, is [8-{8′-2′-H-2′,2′-(PPh_3_)_2_-*closo*-2′,1′,8′-RhC_2_B_9_H_10_}-2-Cp-*closo*-2,1,8-CoC_2_B_9_H_10_] (**3**), the Cp analog of **IV**_CoCp*_, characterised by NMR spectroscopy by analogy with **IV**_CoCp*_ [8]. The presence of two diastereoisomers is evident from the observation of two Cp and two high-frequency C_cage_*H* resonances in the ^1^H NMR spectrum, the latter assigned to the cobaltacarborane cage, and the diastereoisomeric ratio is approximately 1:1.5.

**Figure 3:**
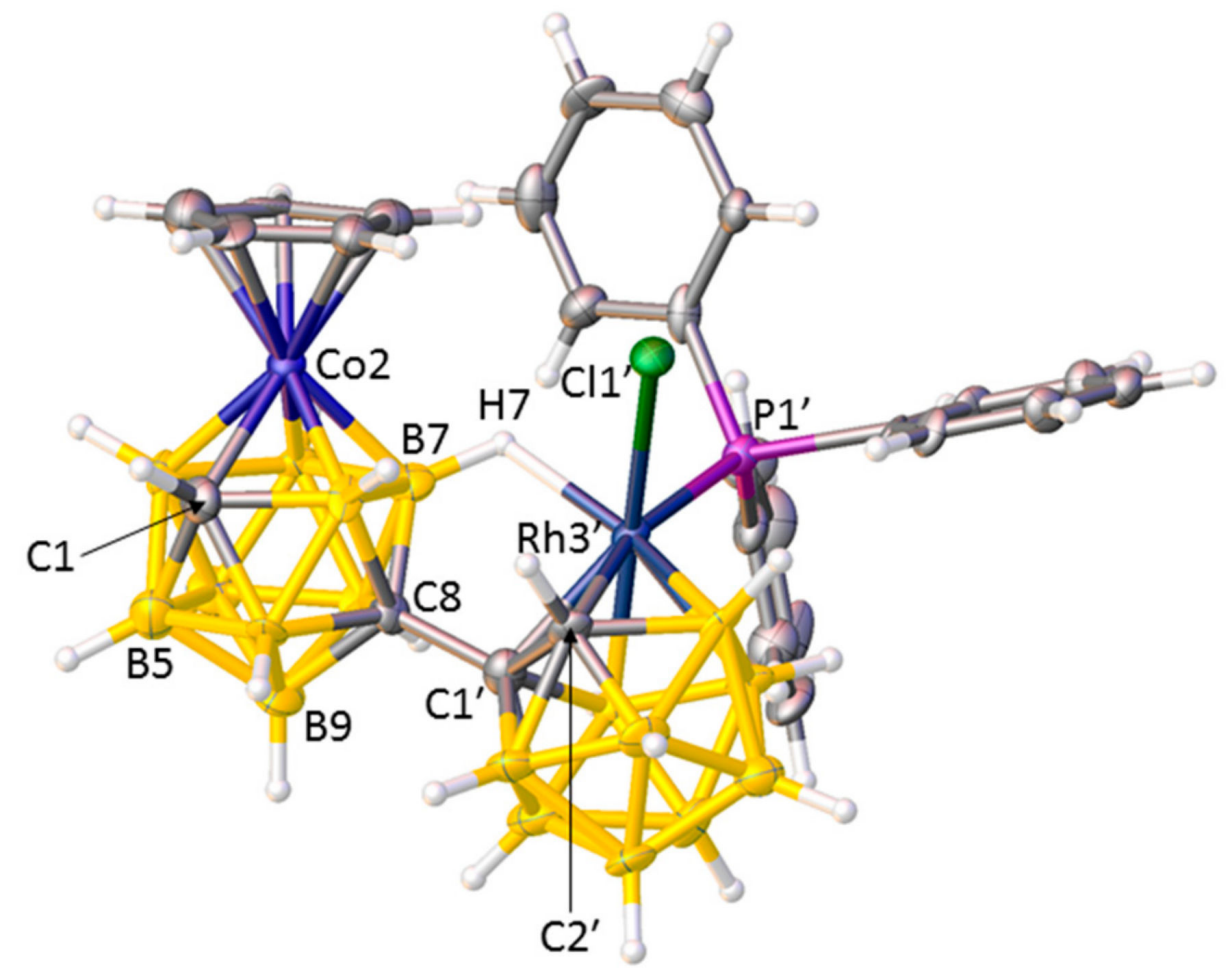
Structure of molecule CD, one of four crystallographically independent molecules of compound **2**. Displacement ellipsoids as in Figure 1. Selected interatomic distances (molecule AB, molecule CD, molecule EF, molecule GH, Å): Ru2−C1 2.048(10), 2.023(11), 2.015(11), 2.030(13); C8−C1′ 1.527(14), 1.530(15), 1.537(15), 1.539(16); Rh3′−C1′ 2,211(9), 2.187(11), 2.214(10), 2.221(12); Rh3′−C2′ 2.236(8), 2.199(9), 2.210(9), 2.190(12); Rh3′−Cl1′ 2.389(2), 2.415(2), 2.395(3), 2.412(2); Rh3′−P1′ 2.222(2), 2.342(3), 2.329(3), 2.347(2); Rh3′−H7 1.92(10), 2.04(11), 2.07(10), 2.12(11); C1′−C2′ 1.633(12), 1.639(13), 1.621(14), 1.649(13).

**Figure 4:**
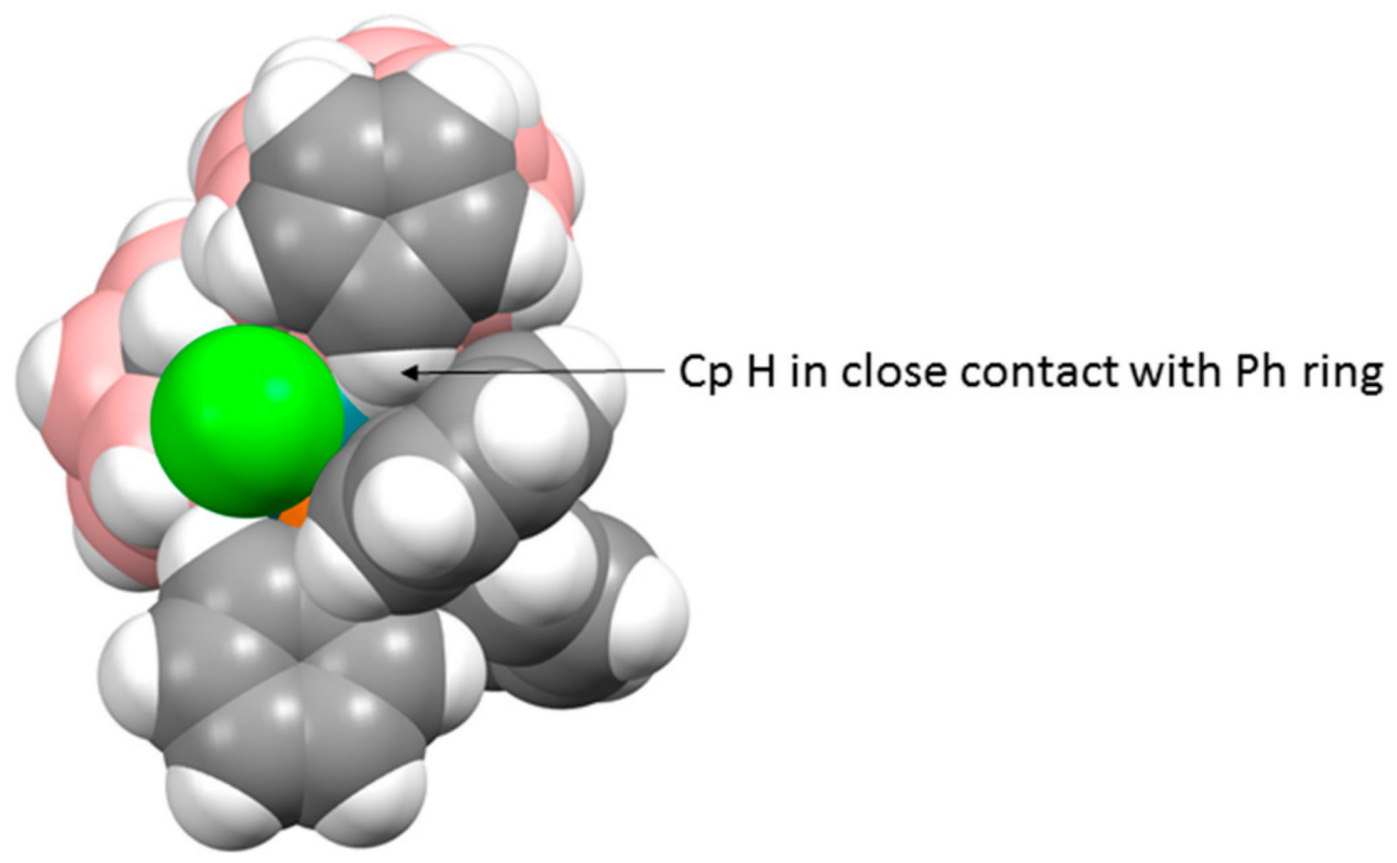
Molecule CD of compound **2** viewed from above in space-filling representation.

However, the major reaction product (15% isolated yield) is [μ_7,8_-(1′,3′−3′-Cl-3′-PPh_3_-*closo*-3′,1′,2′-RhC_2_B_9_H_10_)-2-Cp-*closo*-2,1,8-CoC_2_B_9_H_10_] (**2**), fully analogous to compound **1**. As was the case with **1**, the ^1^H NMR spectrum of **2** suggests that one Ph ring (δ 7.81–7.72 ppm) is in a unique environment, and the results of a crystallographic study support this. Although the structure of compound **2** is not particularly precise, it is unambiguous. There are four crystallographically independent molecules in the asymmetric fraction of the unit cell, again a relatively rare occurrence—somewhat surprisingly, the CCD reports slightly more structures with *Z*′ = 4 than *Z*′ = 3 (4861 c.f. 4654), but in a database of ca. 10^6^ this is still <0.5%. All four independent molecules of **2** are very similar, and a representative example is shown in Figure 3. The structure of compound **2** bears a close similarity to that of compound **1**. One Ph ring stands approximately perpendicular to the plane of the Cp ring, and there are close contacts between one Cp H atom and the Ph ring centroid, ca. 2.6–3.0 Å. A space-filling view of the molecule from above the Cp ring, as shown in Figure 4, is remarkably similar to the analogous representation of compound **1**, and fully consistent with a crowded molecule in which there is contact between the Cp ligand and one Ph ring. Although at room temperature in solution the Cp ring is clearly able to rotate (a singlet observed in the ^1^H NMR spectrum), in the solid state the Ph ring appears locked in conformation.

### 2.3. Control of the Diastereoisomeric Nature of Compounds ***1*** and ***2***

**Figure 5:**
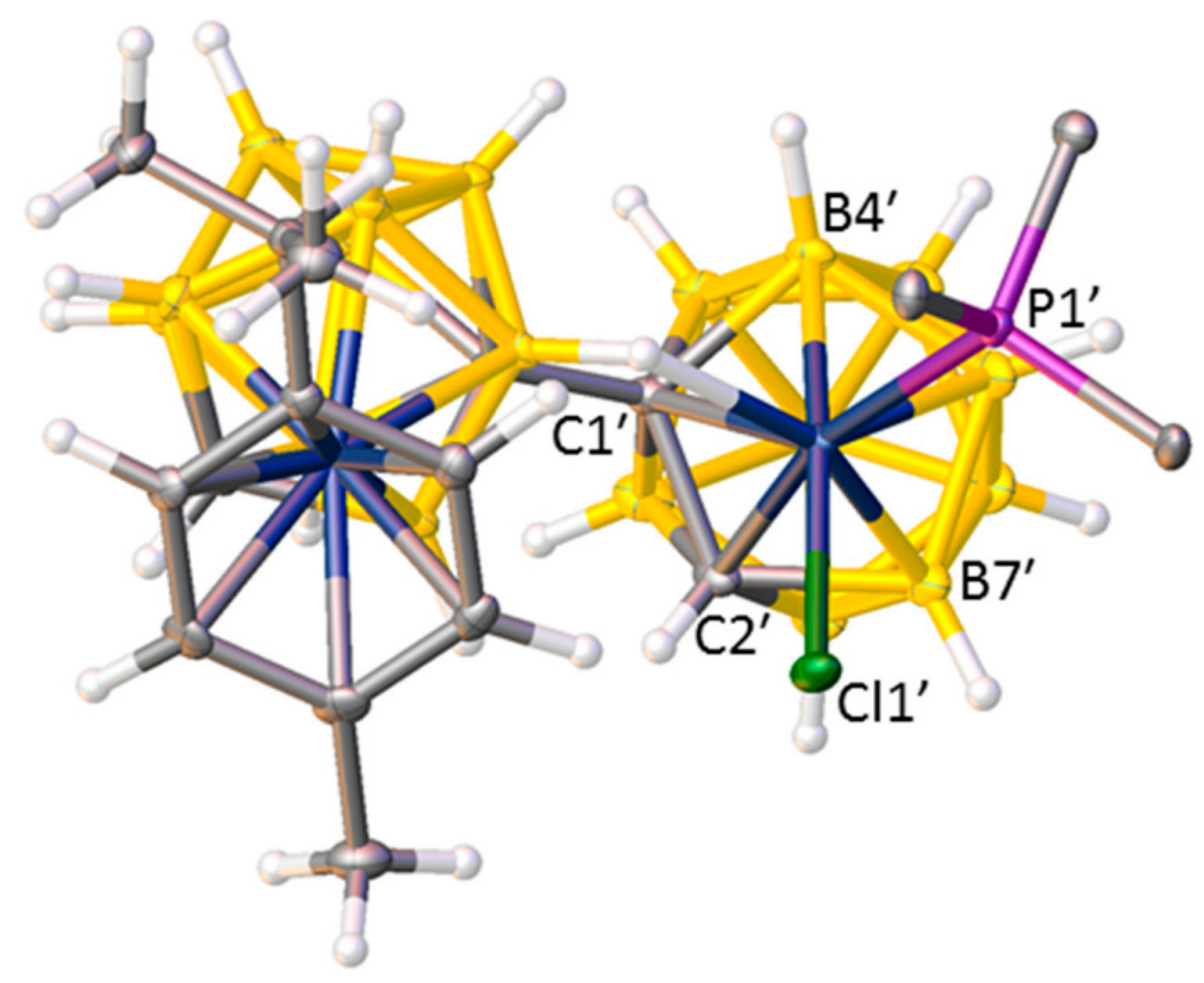
Molecule C of compound **1** from above Rh3′. Only the α atoms of the Ph rings are shown for clarity.

Because anion **III**, the precursor to compounds **1–3**, exists as a mixture of diastereoisomers, products **1–3** would reasonably also be expected to be diastereoisomeric mixtures. Whilst this is true for compound **3**, both **1** and **2** are only isolated as one diastereoisomer. There is no evidence for separation into two diastereoisomers on workup by TLC, nor is there evidence of diastereoisomers in the NMR spectra of **1** and **2**. Both **1** and **2** crystallise with multiple independent molecules in the asymmetric fraction of the unit cell (three for compound **1** and four for compound **2**), but all these multiple molecules are of the same diastereoisomeric form both within and between each compound. Figure 5 shows the structure of molecule **1** viewed from above the Rh atom. The observed diastereoisomer is defined by C at the 2′ position of the rhodacarborane cage and B at the 4′ position, and the “missing” diastereoisomer would be defined by B at 2′ and C at 4′. Thus, identification of the correct diastereoisomer is dependent on the correct assignment of C and B vertices in the structural studies. Whilst distinguishing between C and B vertices crystallographically has traditionally sometimes been challenging, we have recently developed powerful new methods to overcome this problem, specifically the *vertex-centroid distance* (VCD) [12] and *boron-hydrogen distance* (BHD) [13] methods. Both approaches were used for compound **1**, but the relative imprecision of the structural study of **2** meant that cage H atoms could not be reliably refined, restricting the structure of **2** to an analysis by only the VCD method. Nevertheless, in both compounds the non-linking cage C atoms were clearly identified in both cages in all seven crystallographically independent molecules, confirming the same single diastereoisomer in all cases.

We believe that one reason for the complementary diastereoisomer not forming could be due to the preferred *exopolyhedral ligand orientation* (ELO) of metal-ligand fragments in metallacarboranes [14]. It is well established that in a carborane ligand, the C atoms in the open face contribute less to the frontier molecular orbitals of the ligand than the B atoms [15], resulting in the C atoms having a weaker structural trans effect (trans influence). Therefore, the preferred ELO is that in which the exopolyhedral ligand with the strongest structural trans effect lies trans to the cage C atoms. In the case of the rhodacarborane components of **1** and **2**, the strongest exopolyhedral ligand will be PPh_3_, rationalising the ELO observed in the observed diastereoisomer. For the non-observed diastereoisomer, with C atoms at the 1′ and 4′ vertices, the preferred position of the PPh_3_ ligand would be above B7′, trans to the 1′−4′ connectivity. However, the Rh atom is bound to the ruthenacarborane (compound **1**) or cobaltacarborane (compound **2**) cage via the B7H7 unit as part of a Rh3′H7B7C8C1′ cycle that is likely to restrict full orientational freedom of the ligand set on Rh3′. Consequently, the likely outcome for the non-observed diastereoisomer is simply that the PPh_3_ and Cl ligands would effectively exchange places. The observed diastereoisomers are already crowded species, evidenced by the interactions between the *p*-cymene ligand in **1** and the Cp ligand in **2** with one of the phenyl rings of PPh_3_. We anticipate that the crowding in the complementary diastereoisomer, with the PPh_3_ ligand in effectively the same position occupied by the Cl ligand (the green atoms in Figure 2 and Figure 4) in the observed form, would simply be untenable.

## 3. Experimental Section

### 3.1. General Considerations

All experiments were performed under an atmosphere of dry nitrogen using standard Schlenk techniques with some subsequent manipulations and purifications carried out in the air. Tetrahydrofuran (THF) was distilled from sodium/benzophenone, dichloromethane (DCM) from CaH_2_, and petroleum ether (40–60 °C, petrol) from sodium. All solvents were freeze-pump-thawed three times before use. Chloroform-D was stored over 4 Å molecular sieves. [HNMe_3_][8-(7′-*nido*-7′,8′-C_2_B_19_H_11_)-2-(*p*-cymene)-*closo*-2,1,8-RuC_2_B_9_H_10_] ([HNMe_3_]**III**_Ru_) [7], [HNMe_3_][8-(7′-*nido*-7′,8′-C_2_B_19_H_11_)-2-Cp-*closo*-2,1,8-CoC_2_B_9_H_10_] ([HNMe_3_]**III**_CoCp_) [7] and [Rh(PPh_3_)_3_Cl] [16] were prepared according to the literature. All other reagents were purchased from Sigma Aldrich Ltd. (Gillingham, UK) or Alfa Aesar (Heysham, UK) and used without further purification. NMR spectra (Supplementary Materials) were recorded at 298 K using a Bruker AVIII-400 spectrometer (Bruker BioSpin AG, Fallenden, Switzerland), with chemical shifts reported relative to the residual protonated solvent peaks (^1^H) or to external standards (^11^B; BF_3_∙OEt_2_ and ^31^P; H_3_PO_4_).

#### 3.1.1. Synthesis and Characterisation of [μ_7,8_-(1′,3′−3′-Cl-3′-PPh_3_-closo-3′,1′,2′-RhC_2_B_9_H_10_)-2-(*p*-cymene)-closo-2,1,8-RuC_2_B_9_H_10_] (**1**)

[HNMe_3_]**III**_Ru_ (100 mg, 0.179 mmol) was dissolved in THF (20 mL) and the solution cooled to 0 °C before *^n^*BuLi (0.15 mL, 2.5 M, 0.375 mmol) was added dropwise. The resulting yellow solution was stirred at room temperature for 1 h. After freezing at −196 °C, [Rh(PPh_3_)_3_Cl] (170 mg, 0.184 mmol) was added and the reaction mixture was allowed to thaw with stirring overnight at room temperature. Volatiles were removed *in vacuo*, and the residue was dissolved in DCM. Following filtration through a silica plug, the filtrate was purified by preparative TLC (1:1, DCM:petrol) to afford a major brown band (*R*_f_ = 0.42) that was subsequently identified as [μ_7,8_-(1′,3′−3′-Cl-3′-PPh_3_-*closo*-3′,1′,2′-RhC_2_B_9_H_10_)-2-(*p*-cymene)-*closo*-2,1,8-RuC_2_B_9_H_10_] (**1**) (18 mg, 0.020 mmol, 11%). Trace amounts (<1%) of both α and β diastereoisomers of the known species [8-{8′-2′-(*p*-cymene)-*closo*-2′,1′,8′-RuC_2_B_9_H_10_}-2-H-2,2-(PPh_3_)_2_-*closo*-2,1,8-RhC_2_B_9_H_10_], **IV**_Ru_, were also isolated and identified spectroscopically [8].

Compound **1**: ^1^H NMR (400 MHz, CDCl_3_): δ 7.82–7.75 (br m, 5H, C_6_*H*_5_), 7.56–7.45 (m, 10H, C_6_*H*_5_), 6.40–6.36 [m, 2H, CH_3_C_6_*H*_4_CH(CH_3_)_2_], 5.02 (br s, 1H, C_cage_*H*), 4.97–4.92 [m, 2H, CH_3_C_6_*H*_4_CH(CH_3_)_2_], 2.98 [app. sept, 1H, CH_3_C_6_H_4_C*H*(CH_3_)_2_], 2.68 (br s, 1H, C_cage_*H*), 2.28 [s, 3H, C*H*_3_C_6_H_4_CH(CH_3_)_2_], 1.30 [d, 3H, CH_3_C_6_H_4_CH(C*H*_3_)_2_, *J*_H-H_ = 6.97 Hz], 1.22 [d, 3H, CH_3_C_6_H_4_CH(C*H*_3_)_2_, *J*_H-H_ = 6.97 Hz]. ^11^B{^1^H} NMR (128 MHz, CDCl_3_): δ 14.0 (1B), 10.7 (1B), −1.8 to −9.3 multiple overlapping resonances with maxima at −1.8, −4.0, −9.3 (total integral 10B), −18.2 to −19.8 multiple overlapping resonances with maxima at −18.2, −19.8 (total integral 6B). ^31^P{^1^H} NMR (162 MHz, CDCl_3_): δ 31.82 (d, *J*_P-Rh_ = 154.6 Hz).

#### 3.1.2. Synthesis and Characterisation of [μ_7,8_-(1′,3′−3′-Cl-3′-PPh_3_-closo-3′,1′,2′-RhC_2_B_9_H_10_)-2-Cp-closo-2,1,8-CoC_2_B_9_H_10_] (**2**) and [8-{8′-2′-H-2′,2′-(PPh_3_)_2_-closo-2′,1′,8′-RhC_2_B_9_H_10_}-2-Cp-closo-2,1,8-CoC_2_B_9_H_10_] (**3**)

[HNMe_3_]**III**_CoCp_ (100 mg, 0.223 mmol) and [Rh(PPh_3_)_3_Cl] (206 mg, 0.223 mmol) were dissolved in EtOH (50 mL), and the resulting red suspension was heated to reflux for 48 h. Following cooling, the solvent was removed in vacuo and the residue was dissolved in DCM. Following a flash silica plug using DCM:petrol (1:1), spot TLC of the solution using the same eluent revealed a minor yellow band (*R*_f_ = 0.54) and several trace bands. The yellow band was purified by preparative TLC to afford [8-{8′-2′-H-2′,2′-(PPh_3_)_2_-*closo*-2′,1′,8′-RhC_2_B_9_H_10_}-2-Cp-*closo*-2,1,8-CoC_2_B_9_H_10_] (**3**) (12 mg, 0.012 mmol, 5%) as a mixture of diastereoisomers. Due to the small amount of product obtained from the flash silica plug, the baseline was extracted using MeCN and the resulting brown solution evaporated in vacuo. Subsequently, the residue was redissolved in DCM, and spot TLC (DCM:petrol, 7:3) revealed a major brown band (*R*_f_ = 0.38). This was purified by preparative TLC using the same eluent system to yield [μ_7,8_-(1′,3′−3′-Cl-3′-PPh_3_-*closo*-3′,1′,2′-RhC_2_B_9_H_10_)-2-Cp-*closo*-2,1,8-CoC_2_B_9_H_10_] (**2**) (26 mg, 0.033 mmol, 15%).

Compound **3**: ^1^H NMR (400 MHz, CDCl_3_, major diastereoisomer): δ 7.42–7.09 (m, 30H, C_6_*H*_5_), 5.29 (s, 5H, C_5_*H*_5_), 2.49 (br s, 1H, C_cage_*H*), 1.37 (br s, 1H, C_cage_*H*) −8.43 to −8.65 (m, 1H, Rh*H*). ^1^H NMR (400 MHz, CDCl_3_, minor diastereoisomer): δ 7.42-7.09 (m, 30H, C_6_*H*_5_), 5.25 (s, 5H, C_5_*H*_5_), 2.54 (br s, 1H, C_cage_*H*), 1.37 (br s, 1H, C_cage_*H*), −8.43 to −8.65 (m, 1H, Rh*H*). ^11^B{^1^H} NMR (128 MHz, CDCl_3_): δ 0.5 to −8.1 multiple overlapping resonances with maxima at 0.5, −0.8, −5.7, −8.1 (total integral 12B), −12.1 to −21.6 multiple overlapping resonances with maxima at −12.1, −15.5, −21.6 (total integral 6B). ^31^P{^1^H} NMR (162 MHz, CDCl_3_): δ 37.07 (dd, 1P, *J*_P-Rh_ = 109.0 Hz, *J*_P-P_ = 25.8 Hz), 32.58 (d, 1P, *J*_P-Rh_ = 107.0 Hz, *J*_P-P_ = 25.8 Hz).

Compound **2**: ^1^H NMR (400 MHz, CDCl_3_): δ 7.81–7.72 (br m, 5H, C_6_*H*_5_), 7.53–7.44 (m, 10H, C_6_*H*_5_), 5.25 (s, 5H, C_5_*H*_5_), 5.01 (br s, 1H, C_cage_*H*), 2.68 (br s, 1H, C_cage_*H*). ^11^B{^1^H} NMR (128 MHz, CDCl_3_): δ 14.5 (1B), 11.2 (1B), 1.7 (2B), −1.1 (3B), −8.4 (4B), −11.8 (1B), −14.2 (2B), −17.3 (2B), −21.2 (2B). ^31^P{^1^H} NMR (162 MHz, CDCl_3_): δ 32.72 (d, *J*_P-Rh_ = 156.6 Hz).

### 3.2. Crystallographic Studies

Compound **1**, crystal data: Single crystal, C_32_H_49_B_18_ClPRhRu·CH_2_Cl_2_, *M* = 983.61, triclinic, *P*$\bar{1}$, *a* = 12.9437(2), *b* = 18.0162(3), *c* = 31.7106(5) Å, α = 88.7489(13), β = 78.4215(14), γ = 74.3014(15)°, *U* = 6969.9(2) Å^3^, *Z* = 6, *D*_c_ = 1.406 Mg m^−3^, μ = 0.913 mm^−1^, *F*(000) = 2964. A total of 156,262 data to θ_max_ = 27.48° were collected at 110.00(10) K on a Rigaku AFC12 diffractometer using Mo-*K*_α_ *X*-radiation (λ = 0.71073 Å). A total of 31,947 unique reflections (*R*_int_ = 0.0441) were used to solve (using *SHELXS* [17]) and refine (using *SHELXL* [18]) the structure within the *OLEX2* [19] package. *R*_1_ = 0.0358, w*R*_2_ = 0.0889 for data with *I* ≥ 2σ(*I*), *S* (all data) = 1.015, *E*_max_, *E*_min_ = 1.22, −1.56 eÅ^−3^, respectively. The intensity contribution from one CH_2_Cl_2_ of solvation per molecule of **1** was squeezed out [20]. Cambridge Crystallographic Data Centre (CCDC) 1970248.

Compound **2**, crystal data: Single crystal, C_27_H_40_B_18_ClCoPRh·CH_2_Cl_2_, *M* = 872.35, monoclinic, *P*2_1_/*n*, *a* = 31.8941(9), *b* = 12.2982(3), *c* = 41.5839(14) Å, β = 98.710(3)°, *U* = 16122.7(9) Å^3^, *Z* = 16, *D*_c_ = 1.438 Mg m^−3^, μ = 0.946 mm^−1^, *F*(000) = 7008. A total of 122,226 data to θ_max_ = 25.02° were collected at 100(2) K on a Rigaku FR-E+ diffractometer using Mo-*K*_α_ *X*-radiation (λ = 0.71075 Å). A total of 28,472 unique reflections (*R*_int_ = 0.1147) were used to solve and refine the structure, as for compound **1**. *R*_1_ = 0.1012, w*R*_2_ = 0.2134 for data with *I* ≥ 2σ(*I*), *S* (all data) = 1.045, *E*_max_, *E*_min_ = 4.66 (0.95 Å from Rh3G), −1.78 eÅ^−3^ (0.67 Å from Rh3G), respectively. The intensity contribution from one CH_2_Cl_2_ of solvation per molecule of **2** was squeezed out [20]. CCDC 1970249.

For compound **1**, cage C atoms were distinguished from B atoms by application of the VCD and BHD methods [12,13,14]. However, for compound **2**, the fact that cage H atoms except H7 could not be positionally refined restricted us to the VCD method.

Figure 1, Figure 3 and Figure 5 were drawn with *OLEX2* [19]. Figure 2 and Figure 4 were drawn with *Mercury* [21].

## 4. Conclusions

Two examples of species in which a 2,1,8-MC_2_B_9_ metallacarborane is linked to a 3′,1′,2′-RhC_2_B_9_ metallacarborane via a direct C8−C1′ bond and a B7−H7⇀Rh3′ *B*-agostic interaction are reported. Although such compounds could exist as diastereoisomers, only a single, common diastereoisomer is observed both spectroscopically and as a result of crystallographic studies involving a total of seven crystallographically independent molecules. In both species, there is clear evidence of steric congestion between one Ph ring of the PPh_3_ ligand on Rh and the ligand η-bonded to M. Consideration of the preferred exopolyhedral ligand orientation about Rh in the non-observed diastereoisomer suggests that it would be too sterically crowded to form.

## Supplementary Materials

The following are available online. NMR spectra of all new compounds reported. Crystallographic data for the structures reported in this paper have been deposited with the Cambridge Crystallographic Data Centre as supplementary publications nos. CCDC 1970248 and 1970249 (compounds **1** and **2**). Copies of the data can be obtained free of charge upon application to CCDC, 12 Union Road, Cambridge CB2 1EZ, UK (Fax: +44-1223-336033; email: deposit@ccdc.cam.ac.uk or www: http://www.ccdc.cam.ac.uk).

## References

[1] Sivaev I.B., Bregadze V.I. (2019). 1,1′-Bis(*ortho*-carborane)-based transition metal complexes. Coord. Chem. Rev..

[2] Yruegas S., Axtell J.C., Kirlikovali K.O., Spokoyny A.M., Martin C.D. (2019). Synthesis of 9-borafluorene analogues featuring a three-dimensional 1,1′-bis(*o*-carborane) backbone. Chem. Commun..

[3] Wu J., Cao K., Zhang C.-Y., Xu T.-T., Ding L.-F., Li B., Yang J. (2019). Catalytic Oxidative Dehydrogenative Coupling of Cage B−H/B−H Bonds for Synthesis of Bis(*o*-carborane)s. Org. Lett..

[4] Jeans R.J., Chan A.P.Y., Riley L.E., Taylor J., Rosair G.M., Welch A.J., Sivaev I.B. (2019). Arene-Ruthenium Complexes of 1,1′-Bis(*ortho*-carborane): Synthesis, Characterization, and Catalysis. Inorg. Chem..

[5] Thiripuranathar G., Man W.Y., Palmero C., Chan A.P.Y., Leube B.T., Ellis D., McKay D., Macgregor S.A., Jourdan L., Rosair G.M. (2015). Icosahedral metallacarborane/carborane species derived from 1,1′-bis(*o*-carborane). Dalton Trans..

[6] Thiripuranathar G., Chan A.P.Y., Mandal D., Man W.Y., Argentari M., Rosair G.M., Welch A.J. (2017). Double deboronation and homometalation of 1,1′-bis(*ortho*-carborane). Dalton Trans..

[7] Chan A.P.Y., Rosair G.M., Welch A.J. (2018). Heterometalation of 1,1′-bis(*ortho*-carborane). Inorg. Chem..

[8] Chan A.P.Y., Parkinson J.A., Rosair G.M., Welch A.J. (2020). Bis(phosphine)hydridorhodacarborane Derivatives of 1,1′-Bis(*ortho*-carborane) and their Catalysis of Alkene Isomerization and the Hydrosilylation of Acetophenone. Inorg. Chem..

[9] Behnken P.E., Marder T.B., Baker R.T., Knobler C.B., Thompson M.R., Hawthorne M.F. (1985). Synthesis, Structural Characterization, and Stereospecificity in the Formation of Bimetallic Rhodacarborane Clusters Containing Rh-H-B Bridge Interactions. J. Am. Chem. Soc..

[10] Groom C.R., Bruno I.J., Lightfoot M.P., Ward S.C. (2016). The Cambridge Structural Database. Acta Crystallogr. Sect. B Struct. Sci. Cryst. Eng. Mater..

[11] Baker R.T., Delaney M.S., King III R.E., Knobler C.B., Long J.A., Marder T.B., Paxson T.E., Teller R.G., Hawthorne M.F. (1984). Metallacarboranes in Catalysis. 2. Synthesis and Reactivity of Closo Icosahedral Bis(phosphine)hydridorhodacarboranes and the Crystal and Molecular Structures of Two Unusual *closo*-Phosphinerhodacarborane Complexes. J. Am. Chem. Soc..

[12] McAnaw A., Scott G., Elrick L., Rosair G.M., Welch A.J. (2013). The VCD method—A simple and reliable way to distinguish cage C and B atoms in (hetero)carborane structures determined crystallographically. Dalton Trans..

[13] McAnaw A., Lopez M.E., Ellis D., Rosair G.M., Welch A.J. (2014). Asymmetric 1,8/13,2,x-M_2_C_2_B_10_ 14-vertex metallacarboranes by direct electrophilic insertion reactions; the VCD and BHD methods in critical analysis of cage C atom positions. Dalton Trans..

[14] Welch A.J. (2017). What can we learn from the crystal structures of metallacarboranes?. Crystals.

[15] Mingos D.M.P., Forsyth M.I., Welch A.J. (1978). Molecular and Crystal Structure of 3,3-Bis(triethylphosphine)-1,2-dicarba-3-platinadodecaborane and Molecular-orbital Analysis of the “Slip” Distortion in Carbametallaboranes. J. Chem. Soc. Dalton Trans..

[16] Osborn J.A., Wilkinson G., Mrowca J.J. (1990). Chlorotris(triphenylphosphine)rhodium(I) (Wilkinson’s catalyst). Inorg. Synth..

[17] Sheldrick G.M. (2008). A short history of *SHELX*. Acta Crystallogr. Sect. A Found. Crystallogr..

[18] Sheldrick G.M. (2015). Crystal structure refinement with *SHELXL*. Acta Crystallogr. Sect. C Struct. Chem..

[19] Dolomanov O.V., Bourhis L.J., Gildea R.J., Howard J.A.K., Puschmann H. (2009). *OLEX2*: A complete structure solution, refinement and analysis program. J. Appl. Cryst..

[20] Van der Sluis P., Spek A.L. (1990). *BYPASS*: An effective method for the refinement of crystal structures containing disordered solvent regions. Acta Crystallogr. Sect. A Found. Crystallogr..

[21] Macrae C.F., Bruno I.J., Chisholm J.A., Edgington P.R., McCabe P., Pidcock E., Rodriguez-Monge L., Taylor R., van de Streek J., Wood P.A. (2008). *Mercury CSD 2.0*—New features for the visualization and investigation of crystal structures. J. Appl. Cryst..

