# Supplementary material for: Exopolyhedral Ligand Orientation Controls Diastereoisomer in Mixed-Metal Bis(Carboranes)
Source: Molecules. 2020 Jan 24;25(3):519. doi: 10.3390/molecules25030519 (PMC7037510; doi:10.3390/molecules25030519)

Exopolyhedral Ligand Orientation controls Diastereoisomer in Mixed-metal Bis(carboranes)

Antony P. Y. Chan, Georgina M. Rosair and Alan J. Welch

Institute of Chemical Sciences, School of Engineering & Physical Sciences, Heriot-Watt University, Edinburgh Scotland EH14 4AS, UK.

SUPPORTING MATERIALS

^{1}H, $^{11}B\{^{1}H\}$ and $^{31}P\{^{1}H\}$ NMR spectra of all new products.

% H_2O

@ $(CH_3)_2CO$

CH_2Cl_2

* Protio $CDCl_3$

Compound **1**: [μ$_{7,8}$-(1′,3′−3′-Cl-3′-PPh$_3$-*closo*-3′,1′,2′-RhC$_2$B$_9$H$_{10}$)-2-(*p*-cymene)-*closo*-2,1,8-RuC$_2$B$_9$H$_{10}$]

^{1}H NMR ($CDCl_3$):

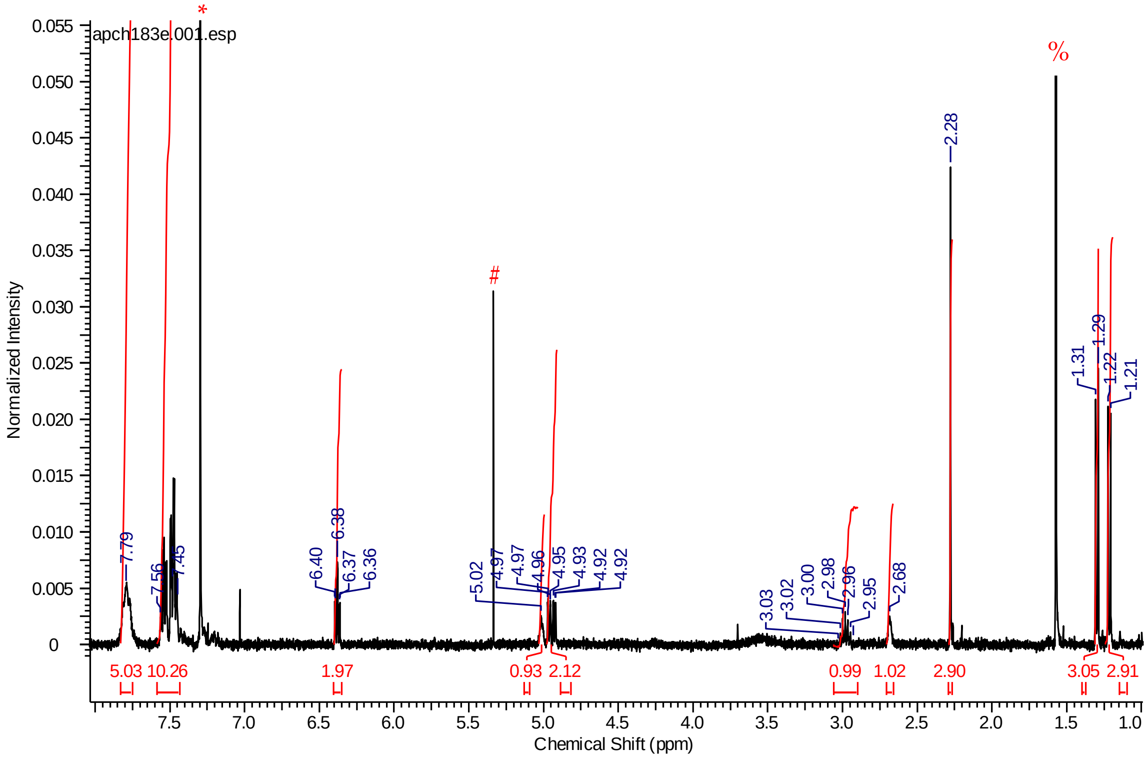

^{11}B{^{1}H} NMR ($CDCl_3$):

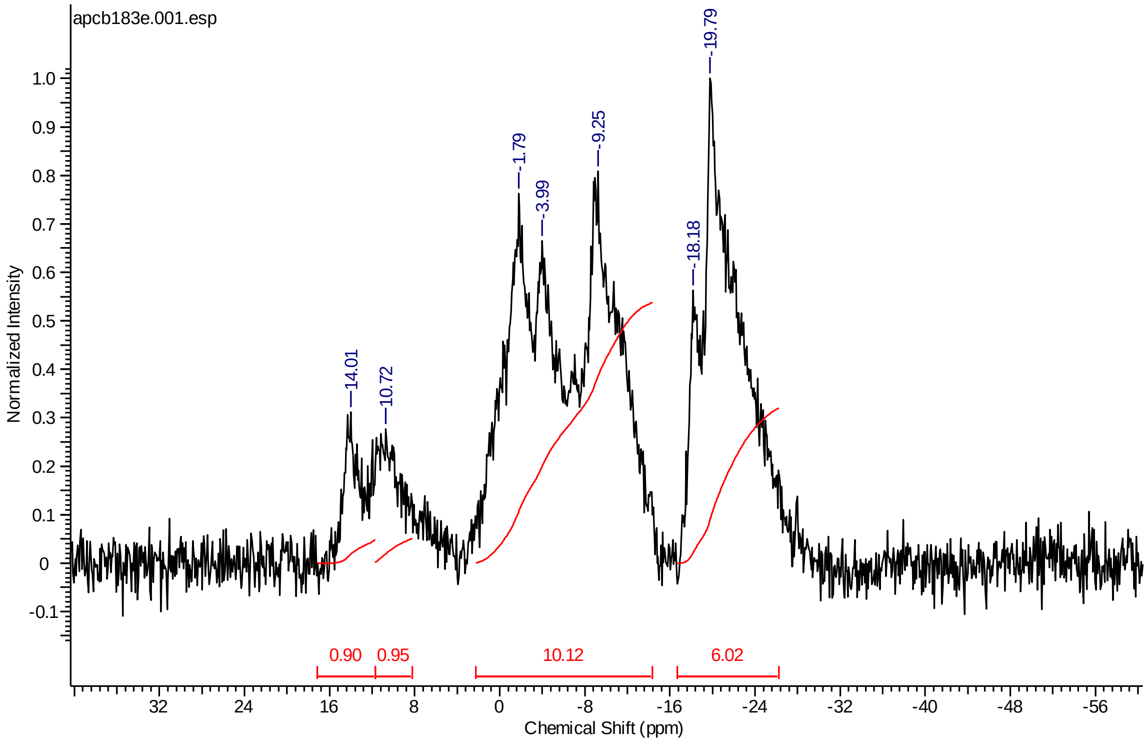

$^{31}P\{^1H\}$ NMR ($CDCl_3$):

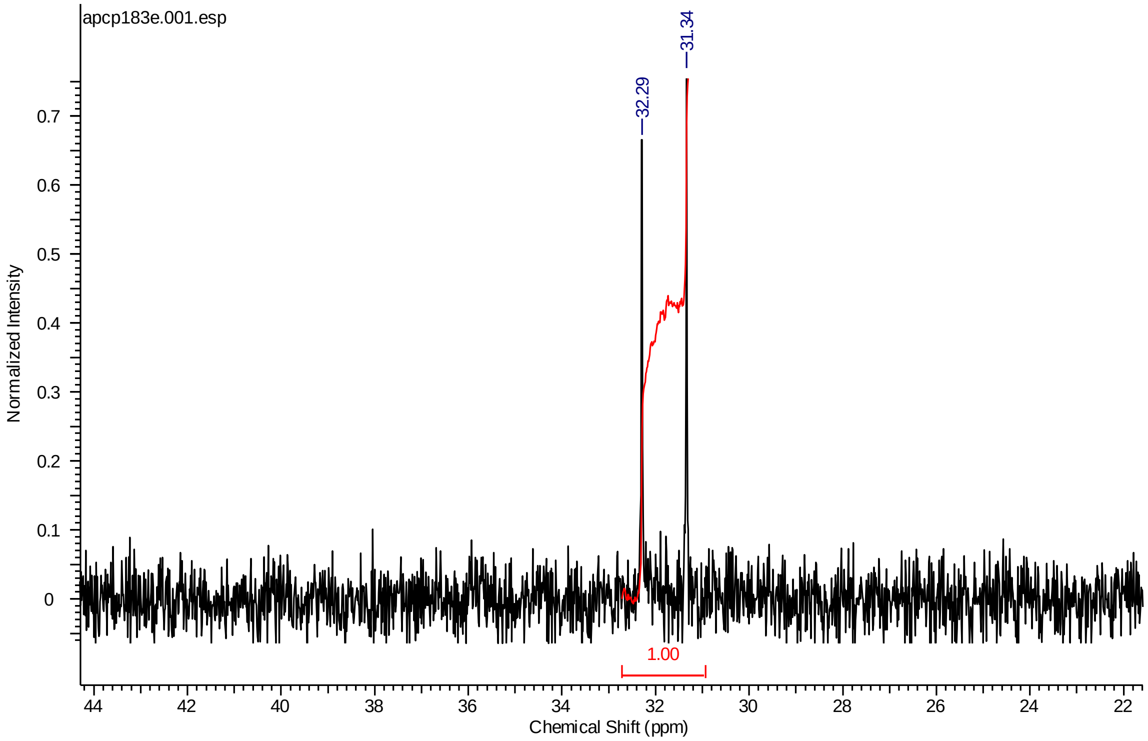

Compound **2**: [μ7,8-(1′,3′–3′-Cl-3′-PPh_3-*closo*-3′,1′,2′-$RhC_2B_9H_{10}$)-2-Cp-*closo*-2,1,8-$CoC_2B_9H_{10}$]

^{1}H NMR ($CDCl_3$):

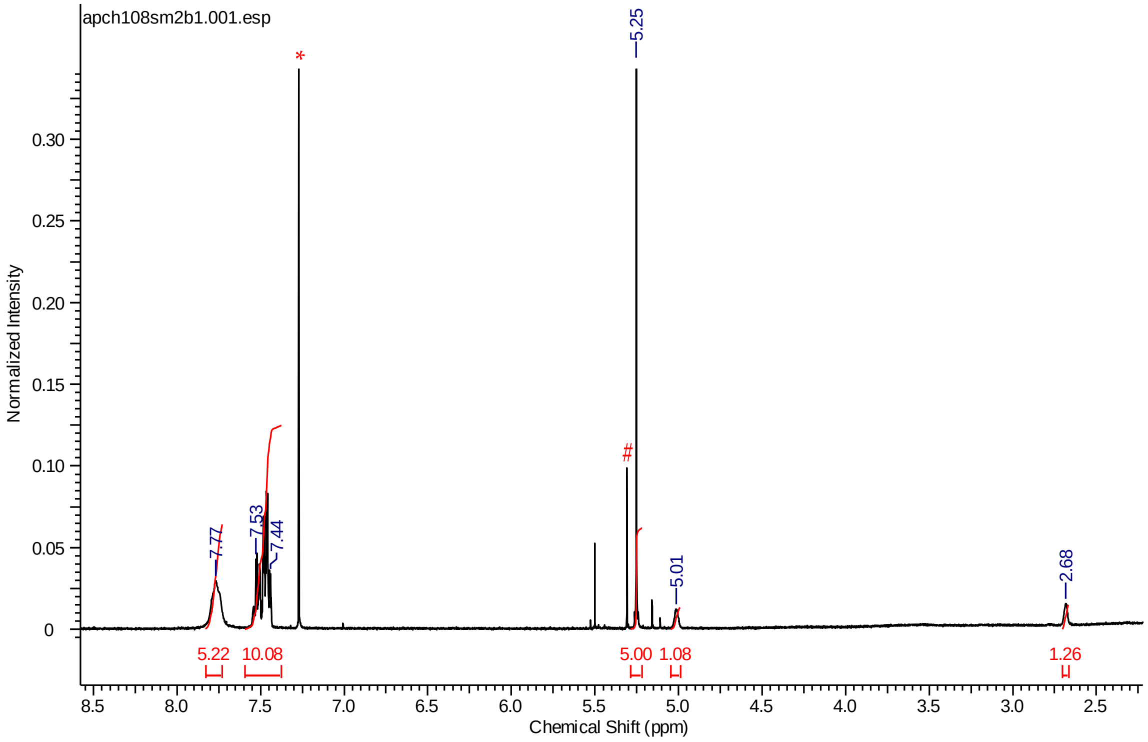

^{11}B{^{1}H} NMR ($CDCl_3$):

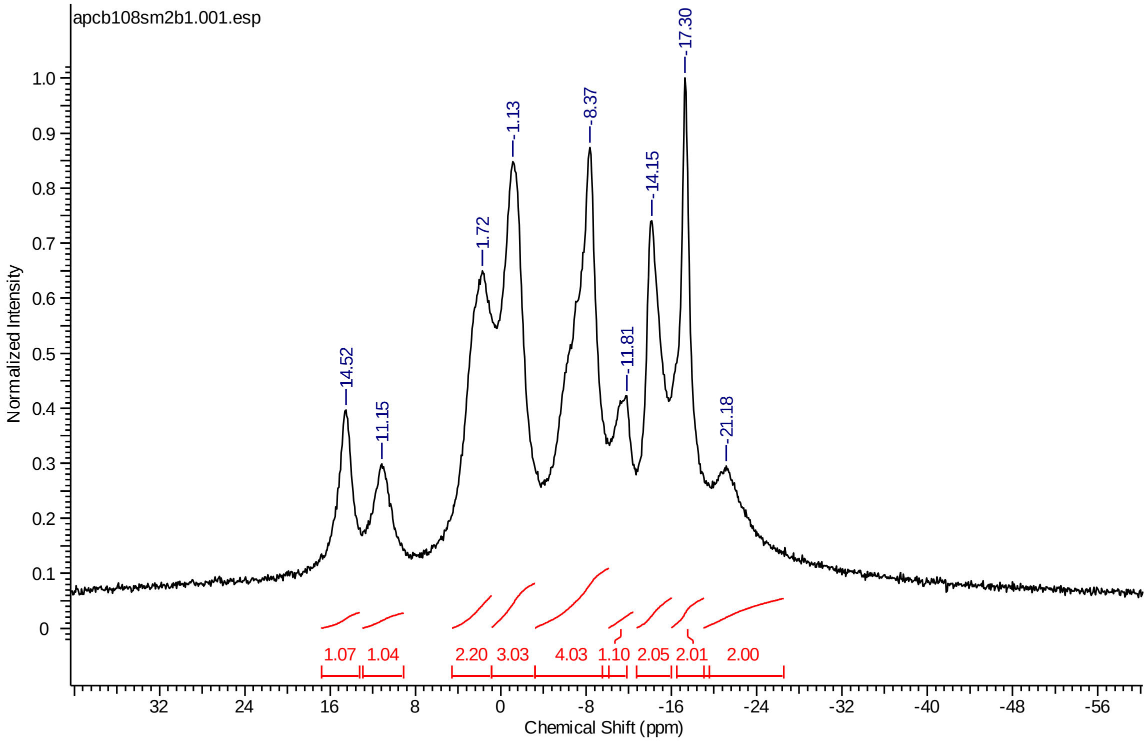

$^{31}P\{^{1}H\}$ NMR ($CDCl_3$):

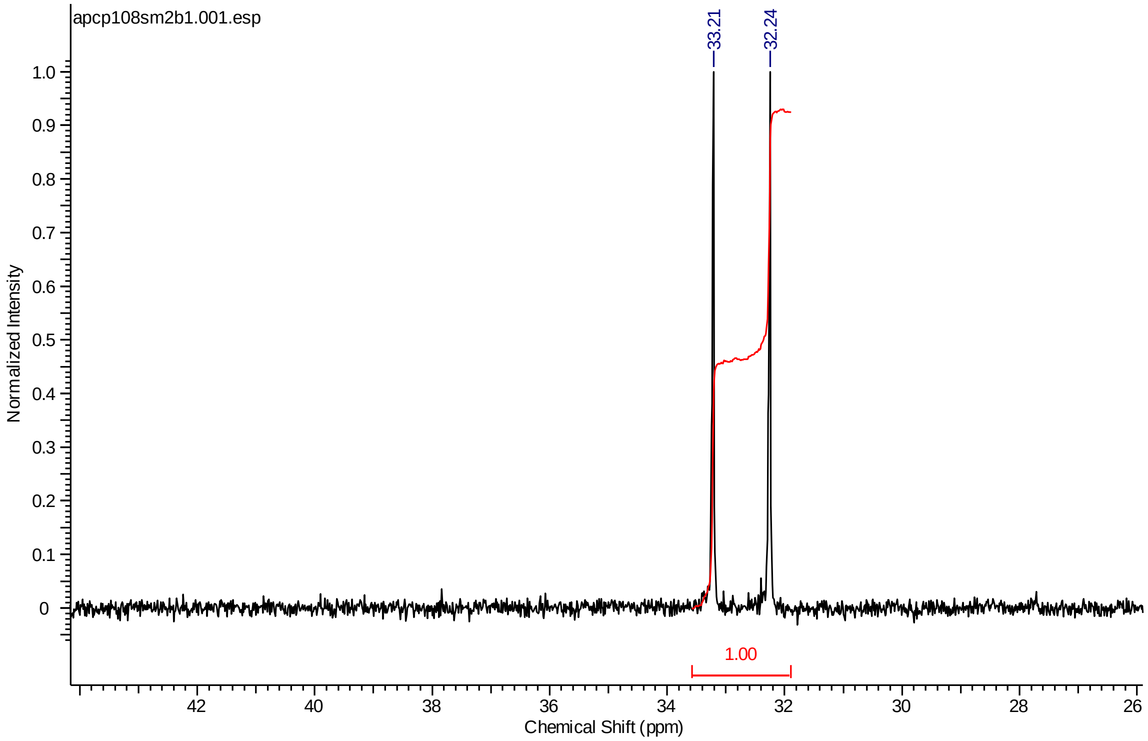

Compound **3**: [8-{8′-2′-H-2′,2′-(PPh_3)$_2$-*closo*-2′,1′,8′-$RhC_2B_9H_{10}$}-2-Cp-*closo*-2,1,8-$CoC_2B_9H_{10}$]

^{1}H NMR ($CDCl_3$):

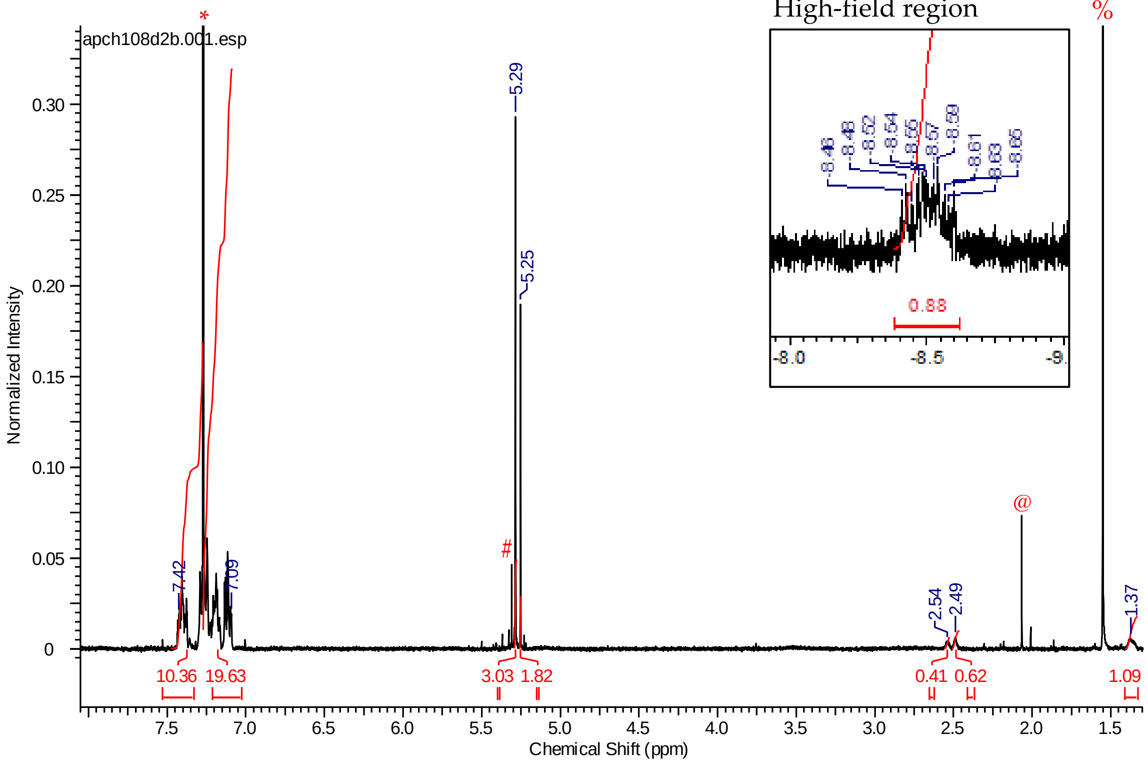

^{11}B{^{1}H} NMR ($CDCl_3$):

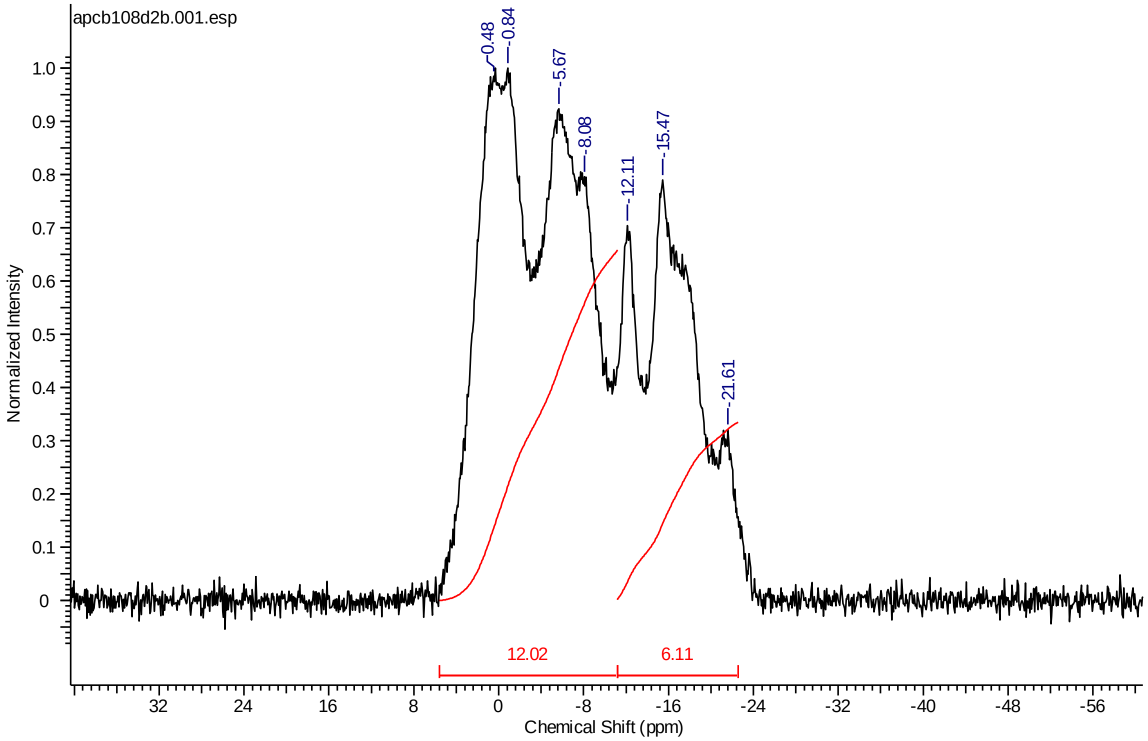

$^{31}P\{^{1}H\}$ NMR ($CDCl_3$):

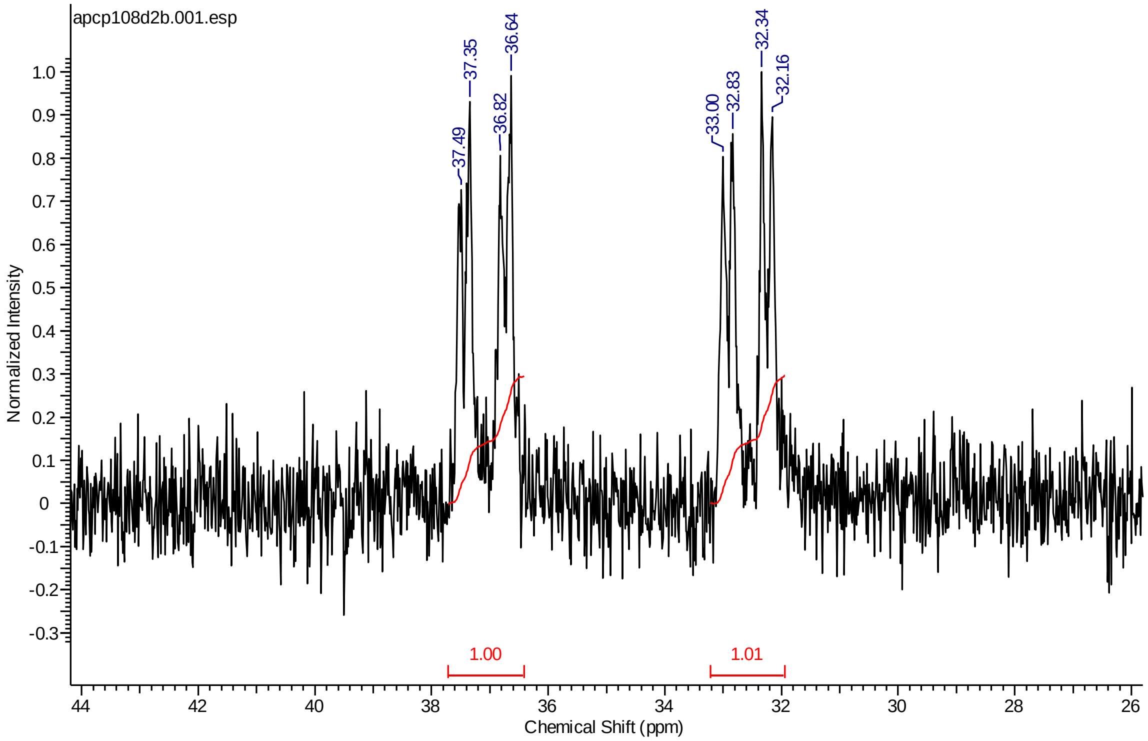

Supplement: Supplementary file 1 [file molecules-25-00519-s001.pdf]
